# Operator independent continuous ultrasound monitoring of diaphragm excursion predicts successful weaning from mechanical ventilation: a prospective observational study

**DOI:** 10.1186/s13054-024-05003-0

**Published:** 2024-07-16

**Authors:** Alexandre Demoule, Quentin Fossé, Alain Mercat, Daniel Bergum, Sara Virolle, Côme Bureau, Marit Mellemseter, Rafaël Guichou, Thomas Similowski, Martin Dres, Satar Mortaza

**Affiliations:** 1 Service de Médecine Intensive - Réanimation (Département R3S), AP-HP, Groupe Hospitalier Universitaire APHP-Sorbonne Université, site Pitié-Salpêtrière, 47-83 Boulevard de L’Hôpital, 75651 Paris Cedex 13, 75013 Paris, France; 2 UMRS1158 Neurophysiologie Respiratoire Expérimentale et Clinique, Sorbonne Université, INSERM, 75005 Paris, France; 3 Service de Réanimation Médicale et Médecine Hyperbare, Centre Hospitalier Régional Universitaire, Angers, France; 4 Department of Intensive Care Medicine, St. Olav University Hospital, Trondheim, Norway; 5 RESPINOR AS, Oslo, Norway; 6 Département R3S, AP-HP, Groupe Hospitalier Universitaire APHP-Sorbonne Université, Site Pitié-Salpêtrière, 75013 Paris, France

**Keywords:** Mechanical ventilation, Weaning, Diaphragm, Ultrasound

## Abstract

**Background:**

In mechanically ventilated patients, diaphragm ultrasound can identify diaphragm weakness and predict weaning failure. We evaluated whether a novel operator-independent ultrasound-based medical device allowing continuous monitoring of the diaphragm (CUSdi) could reliably (1) measure diaphragm excursion (EXdi) and peak contraction velocity (PCVdi), (2) predict weaning outcome, and (3) approximate transdiaphragmatic pressure (Pdi).

**Methods:**

In 49 mechanically ventilated patients, CUSdi was recorded during a 30-min spontaneous breathing trial (SBT), and EXdi and PCVdi were measured. In subgroups of patients, standard ultrasound measurement of EXdi and PCVdi was performed (n = 36), and Pdi derived parameters (peak and pressure time product, n = 30) were measured simultaneously.

**Results:**

The agreement bias between standard ultrasound and CUSdi for EXdi was 0.1 cm (95% confidence interval -0.7–0.9 cm). The regression of Passing-Bablok indicated a lack of systematic difference between EXdi measured with standard ultrasound and CUSdi, which were positively correlated (Rho = 0.84, p < 0.001). Weaning failure was observed in 54% of patients. One, two and three minutes after the onset of the SBT, EXdi was higher in the weaning success group than in the failure group. Two minutes after the onset of the SBT, an EXdi < 1.1 cm predicted weaning failure with a sensitivity of 0.83, a specificity of 0.68, a positive predictive value of 0.76, and a negative predictive value of 0.24. There was a weak correlation between EXdi and both peak Pdi (r = 0.22, 95% confidence interval 0.15 – 0.28) and pressure time product (r = 0.13, 95% confidence interval 0.06 – 0.20). Similar results were observed with PCVdi.

**Conclusions:**

Operator-independent continuous diaphragm monitoring quantifies EXdi reliably and can predict weaning failure with an identified cut-off value of 1.1 cm.

*Trial registration* clinicaltrial.gov, NCT04008875 (submitted 12 April 2019, posted 5 July 2019) and NCT03896048 (submitted 27 March 2019, posted 29 March 2019).

**Supplementary Information:**

The online version contains supplementary material available at 10.1186/s13054-024-05003-0.

## Introduction

Intensive care unit (ICU)-acquired diaphragm weakness occurs in 30 to 60% of patients undergoing mechanical ventilation [1]. Because it impairs respiratory system load capacity balance, diaphragm weakness is associated with weaning failure and increased ICU length of stay and mortality [1–3].

Historically, detecting diaphragm weakness in patients undergoing mechanical ventilation was challenging due to the invasive or expensive nature of available methods, which were confined to expert centers [4, 5]. This limitation spurred the adoption of diaphragm ultrasound as a preferred method. As a non-invasive, safe technique, diaphragm ultrasound offers radiation-free direct visualization and functional assessment of the diaphragm, facilitating broader application in clinical settings [1, 6, 7]. In the last decade, indices of diaphragm motion and contractility such as diaphragm excursion and thickening fraction were shown to reliably identify the outcome of weaning [3, 8, 9], and recent recommendations from the European Society of Intensive Care Medicine agreed on the use of diaphragm excursion to assess diaphragm dysfunction during weaning [10]. More recently, tissue Doppler imaging was applied to the diaphragm to assess tissue motion and velocity [11].

However, shortcomings with current handheld ultrasound methods are the dependence on an expert operator to ensure reliable data collection [12, 13] and continuous monitoring is not feasible, limiting them to providing snapshot data at specific intervals. A new device has been introduced that enables in real-time the measurement of diaphragm's displacement and velocity using ultrasound technology (CUSdi).

We designed a prospective observational study with the following aims: (1) to assess the accuracy and reliability of this continuous ultrasound technique in measuring diaphragm displacement and velocity in comparison with manual operator-dependent standard ultrasound, (2) to explore the correlation between these continuous ultrasound measurements and the simultaneous measure of transdiaphragmatic pressure (Pdi) derived measurements, and (3) to take the advantage of the conduction of a spontaneous breathing trial to determine the performance of the continuous measure of diaphragm excursion and velocity to predict weaning failure.

## Methods

Two studies, A and B, were performed in three ICUs in Europe, two in France (study A, La Pitié-Salpêtrière University Hospital in Paris and University Hospital in Angers) and one in Norway (study B, St. Olav’s University Hospital in Trondheim) between October 2019 and July 2020. Data were pooled for the purpose of this analysis. The study protocols were approved by the local ethics committee in France (Comité de Protection des Personnes Sud-Est 1, n. 2018-13) and Norway (REK midt, n. 2018/941). Patients or next of kin gave written consent to participate. The studies were registered on clinicaltrial.gov, NCT04008875 and NCT03896048.

### Patients

Consecutive patients mechanically ventilated for > 24 h were eligible for inclusion when they met the predefined readiness-to-wean criteria according to our weaning protocol and could therefore undergo a spontaneous breathing trial (SBT). These criteria were as follows: (1) regression or clear improvement of the episode that motivated the institution of mechanical ventilation, (2) FiO_2_ < 50% with positive end-expiratory pressure ≤ 5 cmH_2_O allowing an arterial oxygen saturation ≥ 92%. Non-inclusion criteria were: (1) Richmond Agitation-Sedation Scale (RASS) < -2, (2) noradrenaline dosage > 0.3 μg/kg/min, (3) central or spinal neurological injury involving central ventilatory control or its transmission, (4) invasive mechanical ventilation for more than 14 days, 5) body mass index > 35 kg/m^2^, (6) contraindication to the insertion of the esophageal catheter (i.e., any contraindication to the insertion or change of the gastric tube such as esophageal surgery less than 14 days ago, esophageal varices rupture less than 4 days ago), (7) known neuromuscular disease, (8) administration of neuromuscular blockers less than 24 h ago (excluding succinylcholine for rapid sequence intubation), (9) known hemidiaphragm paralysis or suspicion of hemidiaphragm paralysis (defined as a cupola > 2.5 cm compared to the contralateral cupola on chest X-ray –in the absence of obvious atelectasis, major pleural effusion, pneumothorax or prior lung resection surgery), (10) treatment limitations decision, (11) pregnant woman, (12) age < 18 years or protected adult.

### Measurements

*Continuous ultrasound measurement of diaphragm excursion and velocity (Study A and B)* was performed with the RESPINOR DXT (Diaphragm Excursion Technology, Oslo, Norway), which measures continuously the movement of the right hemidiaphragm in the craniocaudal direction using the upper face of the liver as a proxy for the diaphragm [14]. A variant of the pulsed Doppler principle is used for measuring the motion of the liver and diaphragm. Two sensors were used. The anterior sensor is equipped with an ultrasound beam that emits short wave trains at 2.0 MHz at an angle of 45° and receives echoes from the liver parenchyma in-between the transmissions. The posterior and anterior sensors are fitted with accelerometers, which register their spatial orientation to account for dynamic changes of the beam angle. The posterior and anterior sensors also contain a magnetic distance measurement to compensate for the abdominal movements during respiration. The diaphragm displacement is calculated by summation and displayed and stored at a rate of 200 Hz.

**Fig. 1:**
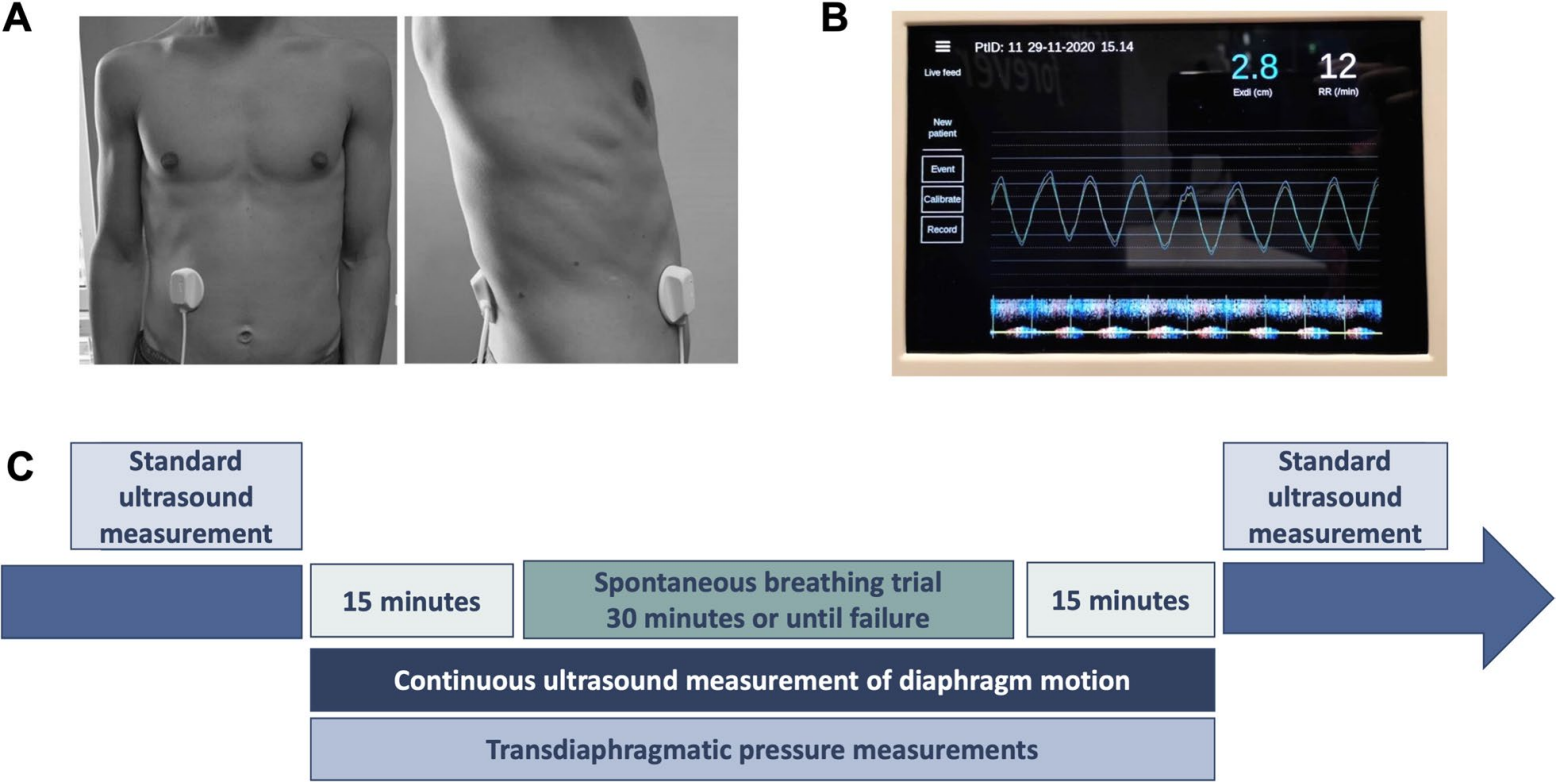
Continuous ultrasound diaphragm excursion and velocity monitoring probe placement and screen. Panel A. *The anterior sensor was placed along the right mid-clavicular line with the upper border of the sensor approximately 2 cm below the lowest rib. The posterior sensor was placed on the back of the patient directly opposite to the anterior sensor*. Panel B. *The screen displays the continuous diaphragm excursion curve as well as the calculated diaphragm excursion in cm (EXdi) and respiratory rate (RR) updated for each breath. The yellow line represents the raw ultrasound signal, and the blue line represents the ultrasound signal compensated for the natural up and down movement of the abdomen during breathing. At the bottom of the screen, a live M-mode plot of diaphragm velocity is displayed. Panel C.* First, a standard ultrasound recording of diaphragm maximal excursion and veloocity (EXdi and PCVdi, 10 cycles each) was performed. Of notice, because of interferences between the ultrasound waves produced by the continuous ultrasound measurement (CUSdi) and the standard ultrasound probes and because the two probes cannot be positioned together at the same location on the abdomen, the two measurements could not be done simultaneously. As such, immediately after the standard ultrasound recording, the sensors for CUSdi were attached and CUSdi recording of diaphragm excursion and velocity was performed. In parallel, the double-balloon catheter was positioned and transdiaphragmatic pressure was continuously measured with. After a 15-min recording of CUSdi and Pdi, the spontaneous breathing trial was initiated, for 30 min or until failure. The recording of CUSdi and Pdi was continued 15 min after the end of the SBT. Right after that, the sensors for CUSdi were removed and a second set of standard ultrasound recording of EXdi and PCVdi (10 cycles each) was immediately performed

The ultrasound beam is centrally cast into a circular polyamide cache that is filled with silicone (Elastosil RT 601, Wacker Chemie AG, Munich), giving the anterior sensor a total diameter of 57 mm (Fig. 1A). The posterior sensor is molded of silicone (Elastosil RT 601), with a diameter of 55 mm and height of 13 mm (Fig. 1A). A 0.4-mm-thick sonolucent double-sided adhesive silicone tape is used to attach the sensors to the patient. The sensors are cabled to a control unit that automatically processes the data in real-time (Fig. 1B) and acts as the user interface and saves the de-identified data for subsequent processing.

The anterior sensor was placed along the right mid-clavicular line with the upper border of the sensor approximately 2 cm below the lowest rib. The posterior sensor was placed on the back of the patient directly opposite the anterior sensor (Fig. 1A).

**Fig. 2:**
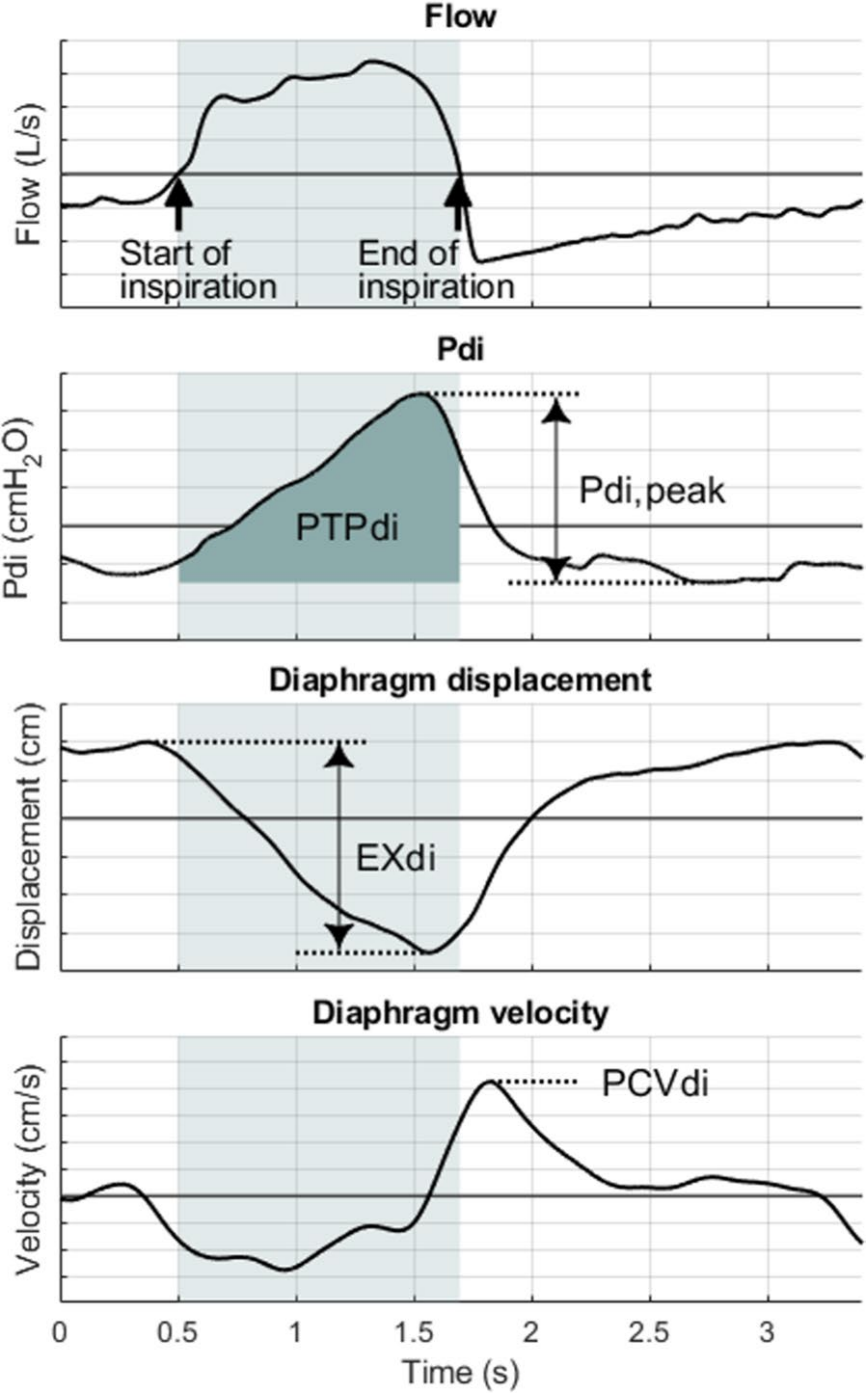
Depiction of the continuous ultrasound measurements of diaphragm excursion and velocity and transdiaphragmatic pressure derived from the time signal from one respiratory cycle

For each breath, the maximal diaphragm excursion (EXdi) and the peak contraction velocity (PCVdi) were measured [11] (Fig. 2).

*Manual standard ultrasound measurement (Study A only)* was performed in each center by the same trained operators using 2–6 MHz broadband curved array transducer [6] connected to a Sparq ultrasound system (Philips Healthcare, Andover, MA, USA). The probe was placed below the right costal margin and directed medially and cephalad. The diaphragm was identified as the hyperechoic linear structure cephalad to the liver. Excursion was measured using M-mode [3, 6, 7] and velocity was measured with tissue Doppler imaging [30]. Images were recorded for subsequent computer-assisted quantitative analysis performed by a trained investigator. EXdi and PCVdi were each measured on 10 separate breaths and the mean of these 10 measurements was reported. Because of interferences between the ultrasound waves produced by the CUSdi and the standard ultrasound probes and because the two probes need to be positioned at the same place on the abdomen, the two measurements could not be performed simultaneously.

*Transdiaphragmatic pressure measurements (Study A only).* Esophageal and gastric pressure (Pes and Pga) were measured using a double-balloon, graduated feeding catheter (NutriVent, Mirandola, Moderna, Italy). The correct position of the esophageal balloon was checked with the occlusion test [15]. Briefly, a dynamic occlusion test was performed to validate esophageal balloon position, allowing the visualization of a corresponding negative deflection in esophageal pressure and airway pressure during inspiratory effort. To validate gastric balloon position, an increase in gastric pressure had to be observed when gently pressing the patient’s abdomen. The esophageal balloon was inflated with 2 mL of air and the gastric balloon was inflated with 4 mL of air. Balloons were connected to a linear differential pressure transducer (MP45, Validyne, Northridge, Calif., USA). Flow was measured using a single use flow sensor (Hamilton Medical, Bonaduz, Switzerland) connected to a pressure transducer (DP45, Validyne, Northridge, CA, USA).

Flow and pressure signals were acquired by a data acquisition system (PowerLab 8/35, AD Instruments, Colorado Springs, CO, USA) at a sampling frequency of 200 Hz. Transdiaphragmatic pressure (Pdi) was continuously obtained by the online subtraction of esophageal pressure from gastric pressure, Pdi = Pga—Pes. Tidal volume was calculated during the SBT by numerical integration over time of the absolute value of the flow signal.

To achieve a concomitant measure of diaphragm displacement and Pdi, an analog timing signal produced by the CUSdi was used to synchronize pressure measurements and continuous ultrasound measurements in post-processing. Signal analyses were performed with the LabChart 8 software (AD Instruments, Colorado Springs, CO) and the MATLAB® 2020b software (Mathworks, Massachusetts, United States).

In each Pdi waveform, we measured Pdi, peak, defined as the difference between the start of the increase in Pdi and the positive peak value of Pdi during inspiration, and the transdiaphragmatic pressure–time product (PTPdi) defined as the area under the transdiaphragmatic pressure curve during inspiration [16] (Fig. 2). Breath cycles were detected by an automatic algorithm in post processing by detecting the zero crossings of the Pdi signal. Artifacts such as coughing, swallowing, or movements, were identified visually on the Pdi signal and discarded from the analysis.

### Study protocol

Figure 1C describes the study protocol. Once the patient was enrolled and prior to the initiation of the SBT, a standard ultrasound recording of EXdi and PCVdi was performed (study A). Immediately after that, the sensors for CUSdi were attached (study A and B) and a CUSdi recording of EXdi and PCVdi was performed. Finally, the double-balloon catheter was positioned (study A). After a 15-min recording of CUSdi and Pdi, the 30-min SBT was initiated. The SBT was performed under T-piece or pressure support ventilation [17, 18]. The SBT was interrupted and considered a failure in case of respiratory rate > 35 breaths/min, arterial oxygen saturation < 90%, heart rate > 140/min (or sustained variation of more than 20% of base value), systolic arterial blood pressure > 180 mmHg or < 90 mmHg, agitation or significant anxiety.

The recording of CUSdi and Pdi was continued 15 min after the end of the SBT, whatever the outcome. Right after that, the sensors for CUSdi were removed and a second set of standard ultrasound recording of EXdi and PCVdi (10 cycles each) was immediately performed (study A).

In the absence of any symptom of poor tolerance mentioned above, the patient was extubated. Patients aged > 60 years or with a chronic underlying cardiac or respiratory disease received either prophylactic non-invasive ventilation or high flow oxygen or both [19, 20]. Weaning failure was defined as patients failing the SBT or passing the SBT but requiring reintubation within the 48 h following extubation. For patients with multiple failed SBT, only their first SBT was considered for the analysis.

Adverse events related to the device such as skin erythema, pain and pruritus were recorded.

Investigators were retrospectively surveyed regarding the ease-of-use of the CUSdi, asking them to rate how they found (a) setting up the system, and (b) placing the sensors, on a scale from 1 to 5 where 1 was very easy and 5 was very difficult, as well as quantifying time spent (a) setting up the system, and (b) placing the sensors, with the following options: 0–2 min, 3–5 min, 6–10 min, 11–20 min, and > 20 min.

### Statistical analysis

Based on previous studies that evaluated EXdi to predict weaning failure [3, 8, 21, 22], we anticipated that weaning failure rate would be 40% in patients with an EXdi < 1.1 cm and 10% in patients with an EXdi > 1.1 cm. With a type I error rate of 0.05 and a power of 0.8, a sample size of 40 patients was needed. Because we anticipated poor signal quality in some patients, we aimed to include 50 patients.

Continuous variables are reported as median (25th–75th percentiles) and categorical variables are expressed as absolute and relative frequency. Continuous variables were compared using a Mann–Whitney U test and categorical variables were compared using a Chi-2 test.

*Comparison between CUSdi and manual standard ultrasound.* The agreement between continuous and standard ultrasound measurements of EXdi and PCVdi (mean of the measurements performed on 10 separate breaths) performed before and after the SBT was evaluated using the method of Bland and Altman [23]. These results were expressed as bias, limits, and 95% confidence interval (CI) of bias. Bias was significant if 0 was not included in the 95%CI. Patients with one measurement outside the limits of agreement defined by the Bland—Altman plot were defined as poor agreement, whereas patients with all measurements within the limits of agreement were defined as good agreement; patient characteristics were compared between the two groups. Continuous and standard ultrasound measurements comparison were achieved using Passing-Bablok linear regression (24) and Spearman correlation.

*Performance of the CUSdi to predict weaning failure.* EXdi and PCVdi were measured 1, 2, 3, 4, 5, 10, 20 and 30 min after the initiation of the SBT (mean of all breath cycles over a 1-min recording, excluding the 5% largest and 5% smallest values as outliers). Receiver operating characteristic (ROC) curves were constructed to evaluate the performance of EXdi and PCVdi to predict weaning failure. Sensitivity, specificity, positive and negative predictive values, and areas under the receiver operating curves (AUC-ROC) were calculated. AUC-ROC were performed to identify optimal cutoff values of EXdi and PCVdi in predicting weaning failure, and these estimates were obtained using bootstrapping with 1000 replications. The best threshold value for each index was determined as the value associated with the best Youden index for the prediction of weaning failure. AUC-ROC were compared using the nonparametric approach of DeLong et al. [25].

*Relationship between CUSdi and simultaneous measure of Pdi.* Peak transdiaphragmatic pressure, PTPdi, EXdi and PCVdi were measured offline every minute during the SBT (trimmed mean of all breath cycles over a 1-min recording, excluding the 5% largest and 5% smallest values as outliers). The relationship between Pdi measurements (Pdi,peak and PTPdi) and CUSdi measurements (EXdi and PCVdi) was assessed with repeated-measures correlation according to the method developed by Bakdash and Marusich [26]. For illustrative purposes, the intra-individual Spearman’s correlations between Pdi measurements and CUSdi measurements were also calculated to highlight the trends within each patient.

Data were analyzed using SPSS (v06, Cary, NC) except for Passing-Bablok regression and Bland–Altman plots that were performed with MedCalc (Mariakerke, Belgium) and for repeated-measures correlation coefficient that were performed with the rmcorr R package (https://cran.r-project.org/web/packages/rmcorr/).

## Results

### Patients' characteristics

**Fig. 3:**
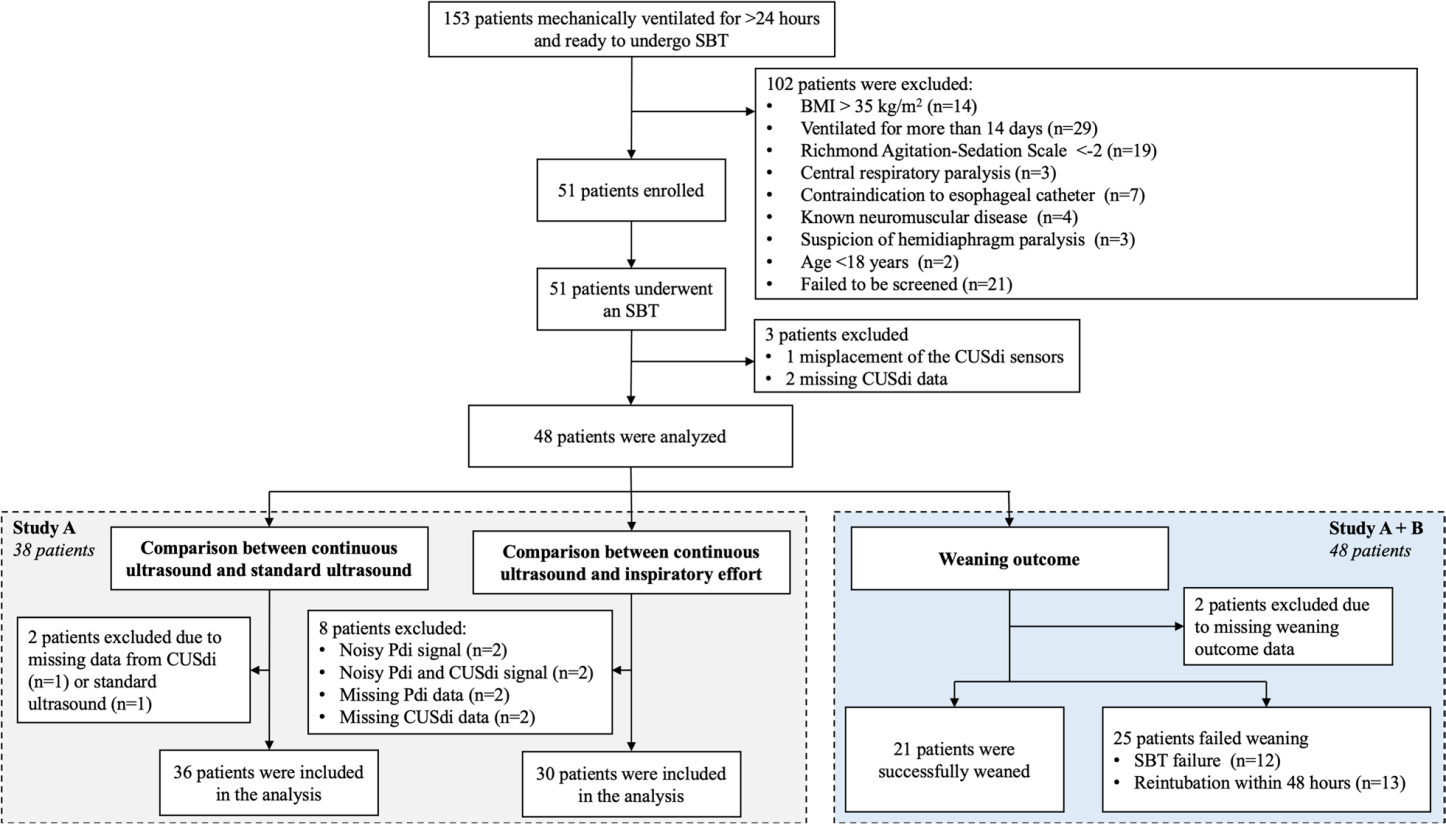
Study flow chart. SBT, spontaneous breathing trial; BMI, body mass index; CUSdi, continuous ultrasound monitoring of the diaphragm; Pdi, trandiaphragmatic pressure

Figure 3 shows the flow chart of Studies A and B. During the study period, 153 patients met the inclusion criteria. Of them, 102 patients had non-inclusion criteria (Fig. 3). Fifty-one patients were enrolled, among whom three patients could not be analyzed (misplacement of the CUSdi sensor in one patient and missing CUSdi data in two patients). Eventually, 48 patients were analyzed, 38 in study A and 10 in study B (Fig. 3). Table 1 describes the main characteristics of the study population. Retrospectively, all the six investigators who performed the experiments found setting up the CUSdi system was very easy or easy. Four out of the six investigators found placing the sensors was easy or very easy and two found it neither easy nor difficult. All the investigators recollected that they spent less than 5 min setting up the system and five out of six recollected that they spent less than 5 min to place the sensors while one investigator recollected that placing the sensors took 6 to 10 min.

**Table 1:** Characteristics of the study population and factors associated with weaning failure by univariate analysis

	All patients^a^ (n = 48)	Weaning success^a^ (n = 21)	Weaning failure^a^ (n = 25)	*P* value
Age, *years*	67 (60–74)	67 (60–73)	66 (60–74)	0.791
Gender, *male, n (%)*	34 (70.8)	14 (67)	18 (72)	0.695
Body mass index, *kg/m*^*2*^	25.8 (23.2–30.3)	23.8 (20.5–28.1)	28.8 (23.8–31.9)	0.006
Charlson comorbidity index	4 (3–5)	4 (2.5–5)	4 (2.5–5.5)	0.639
SAPS II on ICU admission	52 (40–62)	53 (39–62)	52 (40–62)	0.869
Main indication for mechanical ventilation				0.177
Acute respiratory failure, *n (%)*	28 (58)	13 (62)	13 (52)	
Extra-respiratory sepsis, *n (%)*	1 (2)	0 (0.0)	1 (4.0)	
Coma, *n (%)*	10 (21)	6 (29)	4 (16)	
Postoperative/trauma, *n (%)*	1 (2)	0 (0)	1 (4)	
Other, *n (%)*	8 (17)	2 (10)	6 (24)	
Length of MV prior to SBT, *days*	5.6 (3.5–8.6)	6.3 (4.7–9.5)	4.6 (3.1–7.3)	0.178
Two minutes after initiation of the SBT				
Respiratory rate, *breaths/min*	26 (18–31)	26 (17–27)	27 (19–32)	0.180
Tidal volume^*b*^, *mL*	418 (267–539)	411 (204–535)	437 (277–547)	0.453
Tidal volume^*b*^, *mL/kg PBW*	5.5 (3.4–7.3)	5.0 (2.9–8.9)	5.8 (3.6–7.0)	0.435
RSBI^*b*^, *breaths/min/L*	67 (46–98)	74 (61–115)	57 (42–89)	0.322
EXdi, *cm*	1.0 (0.7–1.6)	1.4 (0.9–2.2)	0.8 (0.6–1.0)	0.011
Tidal volume to EXdi ratio^*b*^, *mL/cm*	409 (146–768)	157 (97–683	683 (257–954)	0.133
Diaphragm RSBI^*b*^, *breaths/min/mm*	2.5 (1.1–3.8)	1.8 (0.9–3.3)	2.8 (2.0–5.4)	0.019
Peak diaphragm contraction velocity, *cm/s*	1.4 (0.9–2.3)	2.1 (0.9–2.9)	1.2 (0.8–1.5)	0.019
Pdi,peak^*b*^, *cmH*_*2*_*O*	13.7 (11.0–22.0)	13.5 (11.3–17.0)	15.9 (9.9–25.3)	0.610
Pressure time product of the diaphragm^*b*^, *cmH*_*2*_*O.s/min*	117 (75–186)	108 (75–143)	119 (71–183)	0.704
Outcome variables				
ICU length of stay, *days*	11 (9–15)	10 (7–14.5)	12 (9–14)	0.328
Hospital length of stay, *days*	22.5 (14.0–37.0)	18 (11–29)	25 (16–48)	0.061
Hospital mortality, *n (%)*	8 (17)	3 (14)	5 (20)	0.611

Continuous variables are expressed as median (interquartile) and categorical variables as number (%)

*SAPS* simplified acute physiology score, *ICU* intensive care unit, *MV* mechanical ventilation, *SBT* spontaneous breathing trial, *PBW* predicted body weight, *RSBI* rapid shallow breathing index (respiratory rate/tidal volume), *EXdi* Diaphragm excursion, *Diaphragm RSBI* diaphragm rapid shallow breathing index (respiratory rate/EXdi), *Pdi,peak* peak transdiaphragmatic pressure

^a^Forty-eight patients were included in studies A and B, but information on weaning outcome was missing in two patients (see Results section)

^b^Study A only

### Comparison between continuous ultrasound and standard ultrasound measurements (Study A only)

Comparison between continuous ultrasound and standard ultrasound measurements was performed in 36 of the 38 patients enrolled in study A (CUSdi value was missing in one patient and standard ultrasound value was missing in another patient, Fig. 3), leading to the comparison of 72 pairs of data for EXdi and PCVdi (36 pairs before the initiation of the SBT and 36 pairs after the termination of the SBT).

**Fig. 4:**
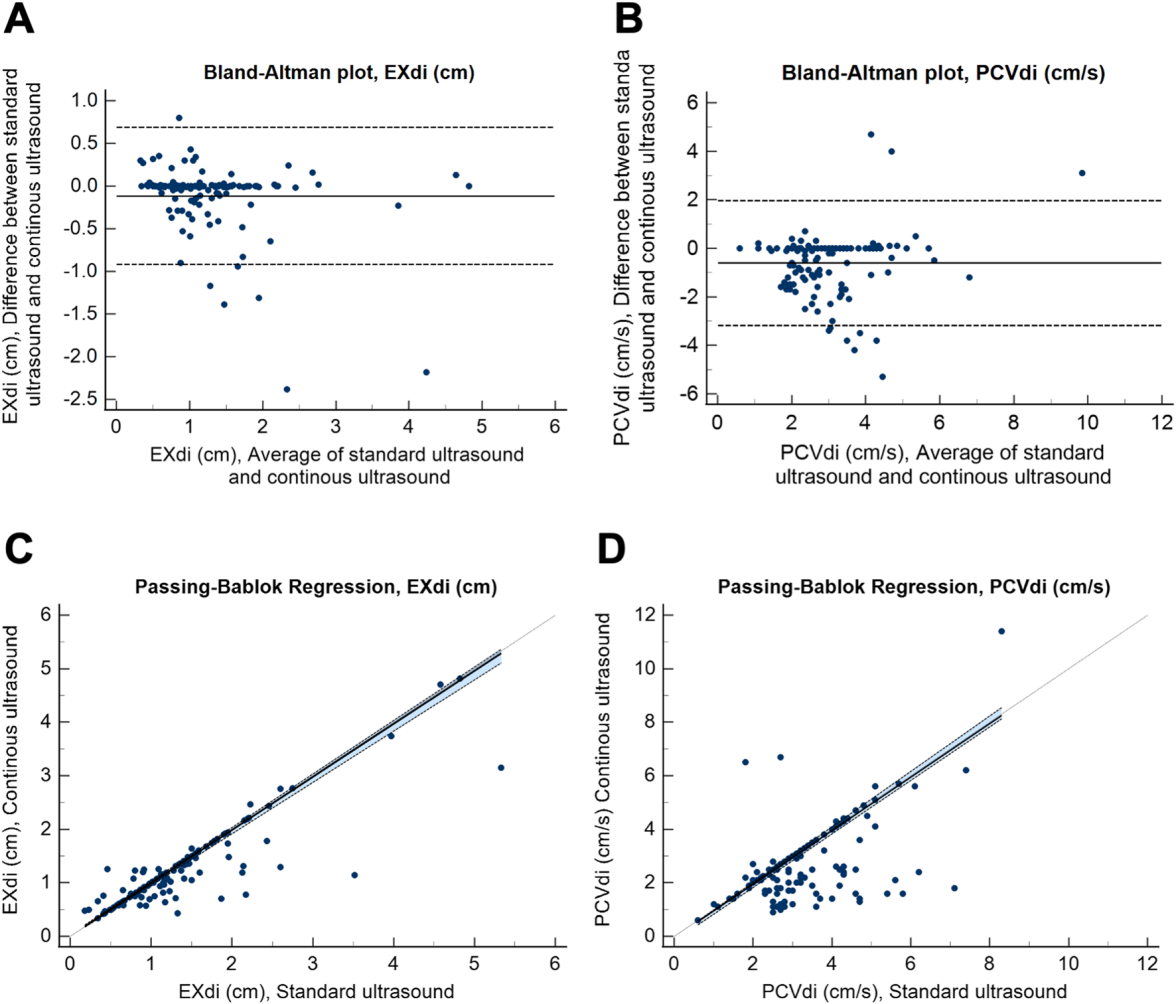
Bland–Altman plot of the agreement for maximal diaphragm excursion (EXdi, Panel A) and peak contraction velocity (PCVdi, Panel B) and Passing-Bablok regression between continuous ultrasound monitoring and standard ultrasound measurement of EXdi (Panel C) and PCVdi (Panel D) measured with continuous ultrasound monitoring and standard ultrasound. Panel A and B shows the difference in EXdi or PCVdi values in the same patients compared with mean EXdi or PCVdi as well as the mean of differences (bias) and limits of agreement (± 2 standard deviation of differences). Regarding the Passing-Bablok regression between continuous ultrasound monitoring and standard ultrasound measurement of EXdi (Panel C), the intercept A of the regression equation was 0.01, 95% CI (0.00–0.03), the slope was 0.99, 95% CI (0.96–1.00) and the CUSUM test for linearity was p = 0.81. Regarding the Passing-Bablok regression between continuous ultrasound monitoring and standard ultrasound measurement of PCVdi (Panel D), the intercept A of the regression equation was -0.05 with a 95% CI (-0.17– -0.05), the slope was 1.00 with a 95% CI (1.00–1.04) and the CUSUM test for linearity was p = 0.27

Diaphragm excursion did not differ between CUSdi and standard ultrasound (respectively 1.1 [0.8–1.4] cm and 1.2 [0.8–1.6] cm). The bias of agreement for EXdi was 0.1 cm with a 95%CI of bias of -0.7 to 0.9 cm. The limits of agreement are shown in Fig. 4A. Patients with poor agreement were taller than those with good agreement and were more likely to be male. The Charlson score did not differ between the two populations, but mild liver disease and diabetes mellitus with end-organ damage were more frequently observed in patients with poor agreement (Additional File 1, Table E1). The Passing-Bablok regression indicated a lack of systematic difference between the two measures (intercept A of the regression Eq. 0.01, 95% CI [0.00–0.03], slope was 0.99, 95% CI [0.96–1.00], CUSUM test for linearity p = 0.81, Fig. 4C) and there was significant positive correlation between EXdi measured with the CUSdi and standard ultrasound (Rho = 0.84, p < 0.001) (Additional File 1, Figure E1).

Peak diaphragm contraction velocity did not differ between CUSdi and standard ultrasound (respectively 2.4 [1.8–3.1] cm/s and 3.0 [2.5–4.1] cm/s). The bias of agreement for PCVdi was 0.6 cm/s with a 95%CI of bias of − 2.0 to 3.2 cm/s. The limits of agreement are shown in Fig. 4B. The Passing-Bablok regression suggested a systematic difference between the two measures but no proportional difference and a linear relationship (intercept A of the regression -0.05, 95% CI [ -0.17– -0.05], slope 1.00, 95% CI [1.00–1.04], CUSUM test for linearity p = 0.27, Fig. 4D) and there was a significant positive correlation between EXdi measured with the CUSdi and standard ultrasound (Rho = 0.44, p < 0.001) (Additional File 1, Figure E1).

### Weaning outcome (Studies A and B)

Weaning outcome was studied in 48 patients included in studies A and B, but information on weaning outcome was missing for two patients. The SBT was performed with T-piece in one patient and with inspiratory pressure support and zero end-expiratory pressure in the remaining 47 patients (median inspiratory pressure level, 7 cmH_2_O).

Twenty-five (54%) of the 46 patients presented weaning failure: 12 failed the SBT while 13 who passed the SBT were extubated and re-intubated within 48 h following extubation. There was no difference in patients characteristics between the weaning success and the weaning failure groups (Table 1).

**Fig. 5:**
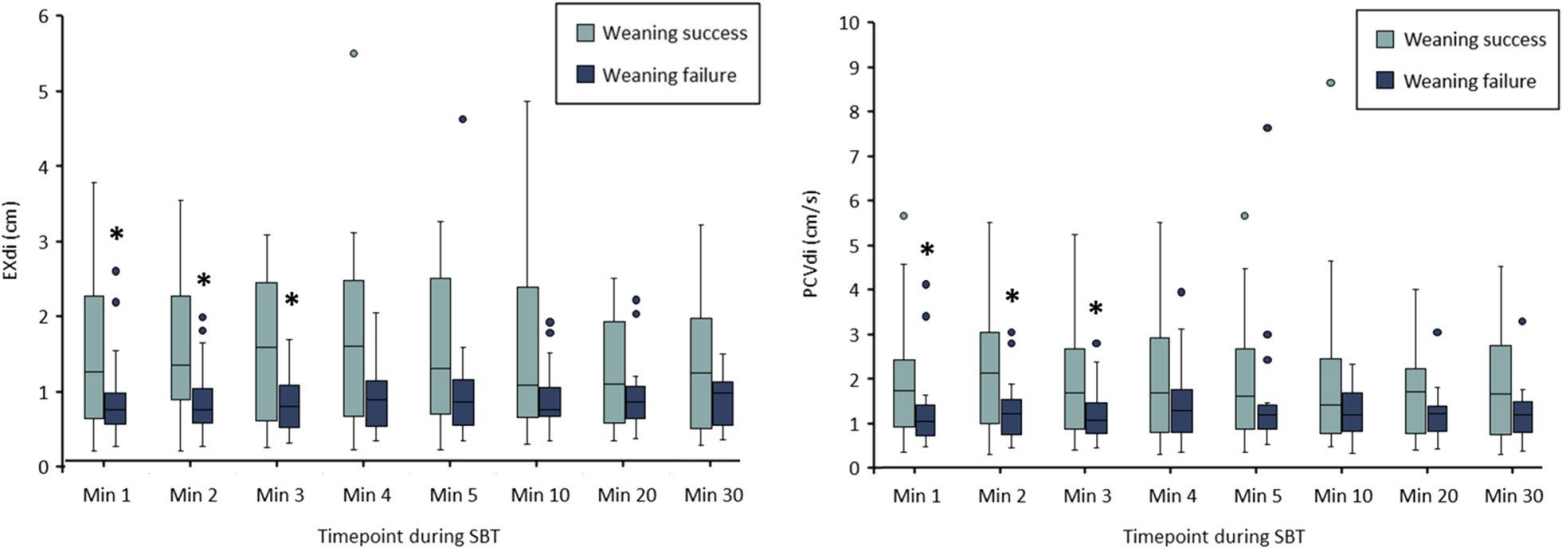
Maximal diaphragm excursion (EXdi) and peak contraction velocity (PCVdi) during the spontaneous breathing trial. EXdi and PCVdi measured 1, 2, 3, 4, 5, 10, 20 and 30 min after the initiation of the SBT in the weaning success group (light boxes) and in the weaning failure (dark blue boxes). Line inside the boxes are median, limits of the boxes are 75th and 25th percentile of the data (interquartile range), and whiskers are 1.5 time the interquartile range. *p < 0.05 vs. failure group

Figure 5 shows EXdi (Panel A) and PCVdi (Panel B) during SBT in weaning success and failure patients. One, two and three minutes after the initiation of the SBT, EXdi was higher in the weaning success group than in the weaning failure group. There was no more difference between the two groups after three minutes of the SBT. An EXdi greater than 1.1 cm 2 min after the onset of the SBT predicted weaning failure with a sensitivity of 0.83 (95%CI 0.61–0.95), a specificity of 0.68 (95%CI 0.44–0.87), a positive predictive value of 0.76 and a negative predictive value of 0.24. A PCVdi greater than 1.5 cm/s 2 min after the onset of the SBT predicted weaning failure with a sensitivity of 0.70 (95%CI 0.47–0.87), a specificity of 0.63 (95%CI 0.38–0.85), a positive predictive value of 0.70 and a negative predictive value of 0.37. Accuracy of EXdi and PCVdi measured with the CUSdi to predict weaning failure 1, 2 and 3 min after the initiation of the SBT is provided in Additional File 1, Table E2.

Two minutes after the initiation of the SBT, there was a significant correlation between EXdi and the actual tidal volume (Rho = -0.403, p = 0.020).

No adverse effect of the device was detected.

### Comparison between continuous ultrasound and inspiratory effort measurements (Study A)

Reliable Pdi or continuous measurements could not be obtained in 8 patients for technical reasons. The comparison between Pdi derived measurements (Pdi,peak and PTPdi) and CUSdi indices (EXdi and PCVdi) was therefore performed in 30 patients (25 pairs per patient in average, varying from 3 to 60).

**Fig. 6:**
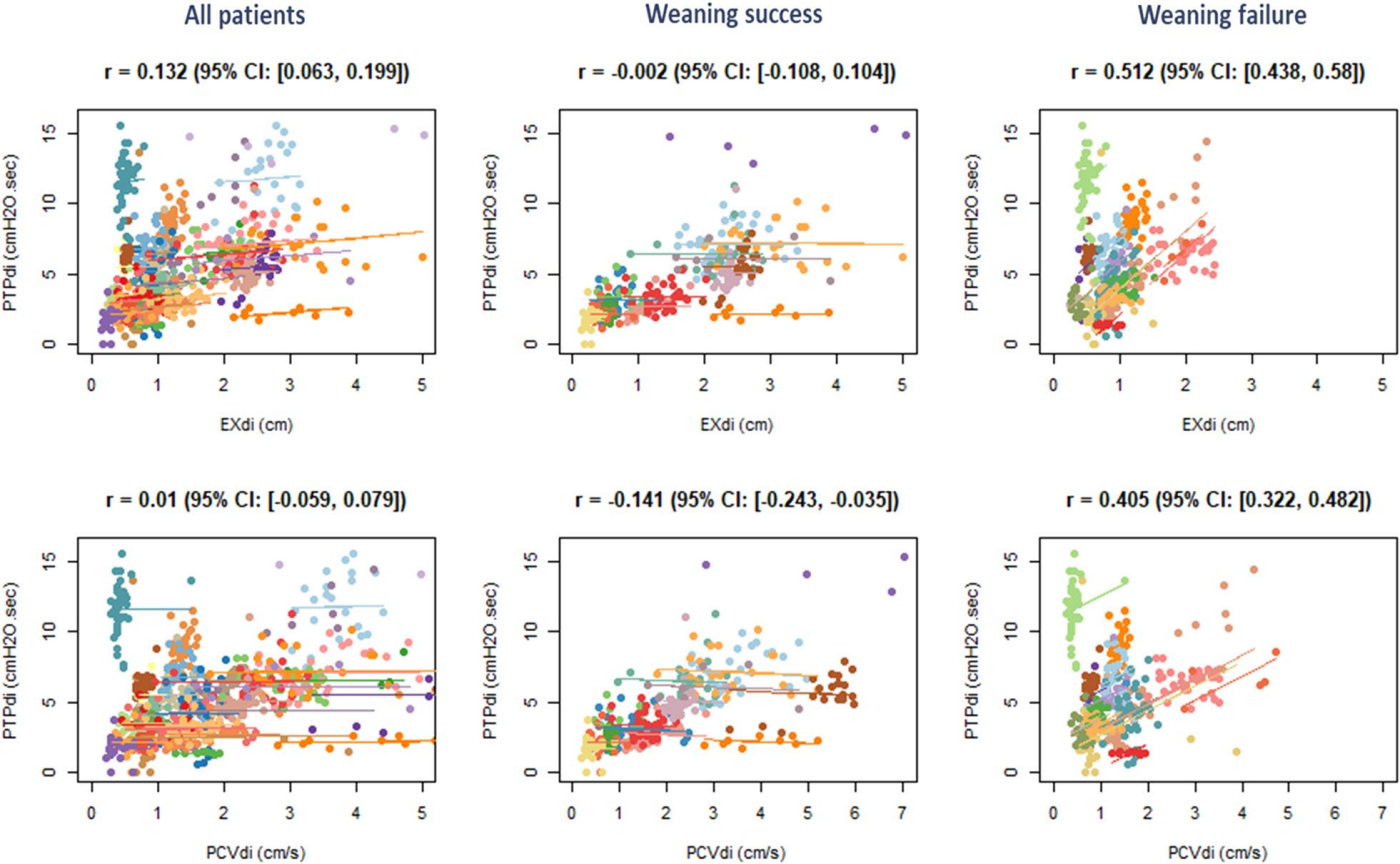
Repeated-measured correlations between pressure time product of the diaphragm (PTPdi), and diaphragm excursion (EXdi, upper panels) and peak contraction velocity (PCVdi, lower panels), in all patients (left panels), in weaning success patients (middle panels) and in weaning failure patients (right panels)

Repeated-measures correlations showed a weak correlation between PTPdi and both EXdi and PCVdi, which was higher in patients who failed weaning than in those who passed (Fig. 6). Repeated-measures correlations also showed a weak correlation between Pdi,peak and both EXdi and PCVdi, which was higher in patients who failed weaning than in those who passed (Additional File 1, Figure E2). Intra-individual Spearman’s correlations are displayed in Additional File 1, Figures E3 and E4 and Tables E3 and E4.

## Discussion

The main and major results are summarized as follows: (1) there was a good agreement between measurements performed by the CUSdi and standard ultrasound, (2) there was a weak correlation between EXdi or PCVdi and Pdi derived measurements (Pdi,peak, and PTPdi), (3) EXdi and, to a lesser extent PCVdi, measured at the second minute of the SBT predicted reliably weaning failure. These results suggest that the CUSdi is a reliable tool to assess EXdi and PCVdi and is useful to predict weaning failure.

The RESPINOR DXT device that records CUSdi is novel, and the first step was to evaluate whether it could reliably measure EXdi and PCVdi, two ultrasound descriptors of diaphragm function. This is the reason why we compared measures performed with the CUSdi and standard ultrasound. Overall, the agreement between CUSdi and standard ultrasound was good, with a low agreement bias. In addition, regarding EXdi, the correlation between the two techniques was excellent (Rho = 0.84). This good agreement between CUSdi and standard ultrasound suggests that, if no experienced operator trained for standard diaphragm ultrasound is available to measure ultrasound derived indices of diaphragm motion, CUSdi can provide reliable ultrasound derived measurements of diaphragm motion. In addition, while the repetition of standard ultrasound examinations is time consuming for operators, this is not the case of CUSdi.

Many studies have evaluated diaphragm excursion and velocity as indices of diaphragm contractility. These studies have shown that diaphragm excursion and velocity were reliable surrogates of diaphragm contractility in spontaneously breathing patients [11, 27–29]. Actually, during pressure support ventilation, diaphragm displacement results from diaphragm intrinsic contractility, but also from the passive displacement resulting from the inflation of lungs by the ventilator. Therefore, active contraction cannot be distinguished from passive displacement due to ventilator inflation [30, 31]. In our study, SBT was performed under pressure support ventilation in all but one patient, with a median inspiratory pressure support level of 7 cmH_2_O and an end-expiratory pressure level of zero cmH_2_O [17, 18]. Although this pressure support level is very low, it elicits diaphragm contractility that is lower than T-piece [32]. We cannot exclude that this low-pressure support level generated a substantial passive diaphragm displacement. This may explain the poor correlation between EXdi and PCVdi and either PTPdi or Pdi, peak. Of notice, such poor correlation between EXdi and PTPdi has been previously reported [33].

The outcome of weaning depends on respiratory system load-capacity balance [34]. To be successful, weaning requires strong and endurant enough respiratory muscles to cope with respiratory system loading [34]. This is why diaphragm function is a major determinant of weaning success in critically ill mechanically ventilated patients [3, 22, 35], diaphragm dysfunction being associated with a higher risk of weaning failure [3, 36]. Diaphragm ultrasonography has become increasingly popular in the ICU because it is easily available at bedside, fast and safe [7]. Recently, experts have produced a consensus-based statement on diaphragm ultrasonography methodology in the ICU [6] and recent recommendations from the European Society of Intensive Care Medicine agreed on the use of diaphragm excursion to assess diaphragm dysfunction during weaning [10]. To date, there is a core of literature showing that decreased EXdi is associated with a higher rate of weaning failure [37]. In a recent meta-analysis that investigated the effectiveness of diaphragm ultrasound to predict the success of weaning from mechanical ventilation, sensitivity of EXdi was 0.80 (95% CI 0.77–0.83) and specificity was 0.80 (95% CI 0.75–0.84). Among the studies included in this meta-analysis, a majority used a cut-off value of 1.0 or 1.1 cm to predict weaning success or failure. We found a similar cut-off value in our study, and with a similar sensitivity but lower specificity (respectively 0.83 and 0.68). Between patients who succeeded in weaning and those who failed, EXdi was significantly different only over the three first minutes of the SBT. Indeed, there was no more significant difference in terms of EXdi between weaning success and failure patients 4, 5, 10, 20 and 30 min after the beginning of the SBT. These might be explained by the drop off in the number of patients who failed weaning, since the SBT had to be terminated earlier in many patients due to failure. Peak contraction velocity of the diaphragm was also effective to discriminate patients who failed from those who succeeded weaning, but to a lesser extent than EXdi. Intriguingly, our results went in the opposite direction compared to what reported Soilemezi et al. [11]. Indeed, we found that PCVdi was lower in patients who failed weaning than in those who passed, while Soilemezi et al. found that it was higher in patients who failed [11]. Differences between the two studies may explain this difference. First, PCVdi was measured at the end of a 30-min SBT in the study by Soilemezi et al., while we measured it at the beginning. Second, it was T-piece SBT, while we performed SBT connected to the ventilator. Third, Soilemezi et al. did not observe a difference between success and failure patients in terms of EXdi, while we did. Fourth, the definition of weaning success differed between the two studies.

Our study appears to be the first report of an automated, continuous ultrasound measurement of diaphragm motion in mechanically ventilated patients. Its main strengths are the demonstration of the reliability of the CUSdi to measure diaphragm excursion and velocity, the effectiveness to predict weaning success or failure and the multicenter design. Our study includes some limitations. First, we performed CUSdi on the right hemidiaphragm because our technology uses the upper face of the liver as a proxy for the diaphragm. Of notice, measuring the EXdi of the left hemidiaphragm is technically more complicated due to air in the stomach. Second, because the anterior sensor is placed on the abdomen, which moves along the diaphragm during inspiration, it may underestimate diaphragm motion. This is the reason why we also used a posterior sensor that contains a magnetic system and a 3-axis accelerometer to compensate actively for anteroposterior abdominal movements during inspiration. Third, for technical reasons, CUSdi and standard ultrasound measurements could not be performed simultaneously, first because of interferences between the ultrasound waves produced by the two probes (CUSdi and standard ultrasound) and second because the two probes should be positioned at the same place on the abdomen, which is not possible. To make sure that the two ultrasound examinations would be performed in comparable conditions, we took two precautions. First, the two ultrasound examinations (CUSdi and standard ultrasound) were performed right after each other, to avoid any change of state between the two measurements. Second, they were performed in two steady states or quasi-so, the first time before the initiation of the SBT and the second time after the SBT, with ventilator settings back to normal. We cannot rule out that outliers may be explained by changes in diaphragm activity between the two recordings. Fourth, our sample size was limited and only three centers participated in the study. A larger study involving more centers is needed before generalizing our results. A head-to-head comparison between EXdi measured with CUSdi and other predictors of weaning success like the RSBI is needed to establish the potential additional value of EXdi to predict weaning failure [38].

## Conclusions

The Operator-independent CUSdi quantifies diaphragm excursion and peak velocity similar to standard ultrasound and can reliably predict weaning failure with a defined cut-off value of 1.1 cm, which is consistent with the literature. The technology has a good safety profile as no adverse device effects have been reported. First, it allows the measurement of ultrasound derived indices of diaphragm motion by operators that are not trained for diaphragm ultrasound. Second, these measures can be performed continuously and repeatedly. A larger prospective multicenter study is also needed to evaluate the generalizability of our results.

### Supplementary Information

**Additional file 1**. Online Supplement.

## Data Availability

The datasets used and/or analyzed during the current study are available from RESPINOR.

## Abbreviations

ICU: Intensive care unit
OR: Odds ratios
CI: Confidence intervals
CUSdi: Continuous ultrasound measurement of diaphragm displacement and velocity
Pdi: Transdiaphragmatic pressure
SBT: Spontaneous breathing trial
EXdi: Maximal diaphragm excursion
PCVdi: Peak diaphragm contraction velocity
Pdi peak: Difference between the start of the increase in transdiaphragmatic pressure and the positive peak value of transdiaphragmatic pressure during inspiration
PTPdi: Transdiaphragmatic pressure–time product
