# Supplementary material for: Operator independent continuous ultrasound monitoring of diaphragm excursion predicts successful weaning from mechanical ventilation: a prospective observational study
Source: Crit Care. 2024 Jul 16;28:245. doi: 10.1186/s13054-024-05003-0 (PMC11253384; doi:10.1186/s13054-024-05003-0)
Supplement: Supplementary file 1 — Additional file 1. Online Supplement. [file 13054_2024_5003_MOESM1_ESM.pdf]

Online Supplement

- **Table E1.** Factors associated with agreement between continuous ultrasound monitoring and standard ultrasound measurement of diaphragm excursion by univariate analysis.

- **Figure E1.** Spearman's correlation between continuous ultrasound monitoring and standard ultrasound measurement of diaphragm excursion (EXdi, Panel A) and peak contraction velocity of the diaphragm (PCVdi, Panel B) measured with continuous ultrasound monitoring and standard ultrasound.

- **Table E2.** Accuracy of maximal diaphragm excursion and peak contraction velocity to predict weaning failure during 1, 2 and 3 minutes after the onset of the spontaneous breathing trial.

- **Figure E2.** Repeated-measures correlations between peak transdiaphragmatic pressure (Pdi,peak), and diaphragm excursion (EXdi, upper panels) and peak contraction velocity (PCVdi, lower panels), in all patients (left panels), in weaning success patients (middle panels) and in weaning failure patients (right panels).

- **Figure E3.** Intra-individual Spearman's correlations between transdiaphragmatic pressure-time product (PTPdi), and diaphragm excursion (EXdi, upper panels) and peak contraction velocity (PCVdi, lower panels), in all patients (left panels), in weaning success patients (middle panels) and in weaning failure patients (right panels). Solid line signifies the linear regression line.

- **Figure E4.** Intra-individual Spearman's correlations between peak transdiaphragmatic pressure (Pdi,peak), and diaphragm excursion (EXdi, upper panels) and peak contraction velocity (PCVdi, lower panels), in all patients (left panels), in weaning success patients (middle panels) and in weaning failure patients (right panels). Solid line signifies the linear regression line.

- **Table E3.** Strength of intra-individual Spearman's correlation between transdiaphragmatic pressure-time product (PTPdi) or peak transdiaphragmatic pressure (Pdi,peak) and diaphragm excursion (EXdi) or peak contraction velocity (PCVdi).

- **Table E4.** Direction of intra-individual Spearman's correlation between transdiaphragmatic pressure-time product (PTPdi) or peak transdiaphragmatic pressure (Pdi,peak) and diaphragm excursion (EXdi) or peak contraction velocity (PCVdi).

Table E1. Factors associated with agreement between continuous ultrasound monitoring and standard ultrasound measurement of diaphragm excursion by univariate analysis.

	Good agreement (n = 30)	Poor agreement (n = 6)	P value
Age, *years*	67 (60-73)	70 (53-75)	0.734
Gender, *male*, *n (%)*	19 (63)	6 (100)	0.075
Height, m	1.70 (1.62-1.76)	1.79 (1.73-1.90)	0.022
Weight, kg	74 (63-90)	77 (67-105)	0.610
Body mass index, kg/m^2	25.2 (23.0-30.2)	25.6 (19.6-29.8)	0.581
SAPS II on ICU admission	53 (40-62)	60 (50-81)	0.316
Charlson comorbidity index	4 (3-6)	4 (2-5)	0.519
Myocardial infarction, *n (%)*	2 (7)	0 (0)	0.515
Congestive heart failure, *n (%)*	3 (10)	1 (17)	0.635
Peripheral vascular disease, *n (%)*	7 (23)	0 (0)	0.317
History of cerebrovascular accident, *n (%)*	4 (13)	0 (0)	0.343
Dementia, *n (%)*	2 (7)	0 (0)	0.515
Chronic obstructive pulmonary disease, *n (%)*	10 (33)	3 (50)	0.438
Connective tissue disease, *n (%)*	1 (3)	0 (0)	0.650
Peptic ulcer disease, *n (%)*	1 (3)	0 (0)	0.650
Mild liver disease, *n (%)*	1 (3)	2 (33)	0.015
Moderate to severe liver disease, *n (%)*	0 (0)	0 (0)	NA
Uncomplicated diabetes mellitus, *n (%)*	6 (20)	0 (0)	0.230
Diabetes mellitus with end-organ damage, *n (%)*	0 (0)	1 (17)	0.023
Hemiplegia, *n (%)*	0 (0)	0 (0)	NA
Moderate to severe chronic kidney disease, *n (%)*	4 (13)	0 (0)	0.343
Localized solid tumor, *n (%)*	1 (3)	0 (0)	0.650
Metastatic solid tumor, *n (%)*	2 (7)	0 (0)	0.515

Leukemia, *n (%)*	1 (3)	0 (0)	0.650
Lymphoma, *n (%)*	1 (3)	0 (0)	0.650
AIDS, *n (%)*	0 (0)	0 (0)	NA

Continuous variables are expressed as median (interquartile) and categorical variables as number (%).

SAPS, simplified acute physiology score; NA, not applicable.

Figure E1. Spearman correlation between continuous ultrasound monitoring and standard ultrasound measurement of diaphragm excursion (EXdi, Panel A) and peak contraction velocity of the diaphragm (PCVdi, Panel B) measured with continuous ultrasound monitoring and standard ultrasound.

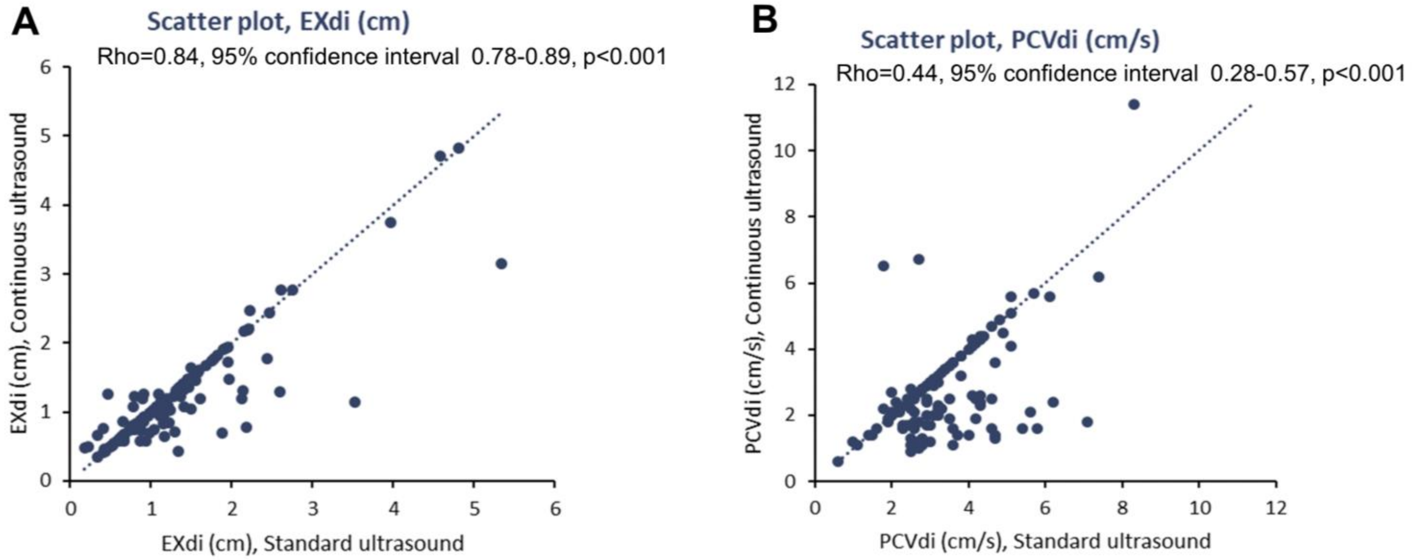

Table E2. Accuracy of maximal diaphragm excursion and peak contraction velocity to predict weaning failure during 1, 2 and 3 minutes after the onset of the spontaneous breathing trial.

Time point	Threshold	AUC (95% CI)	p	Sensitivity (95% CI)	Specificity (95% CI)	PPV	NPV
**Maximal diaphragm excursion**							
Minute 1	10.25 mm	0.71 (0.54-0.88)	0.021	0.82 (0.60-0.95)	0.68 (0.44-0.87)	0.750	0.235
Minute 2	11.25 mm	0.73 (0.57-0.90)	0.011	0.83 (0.61-0.95)	0.68 (0.44-0.87)	0.760	0.235
Minute 3	10.75 mm	0.70 (0.52-0.88)	0.030	0.74 (0.52-0.90)	0.56 (0.31-0.79)	0.680	0.375
**Peak contraction velocity of the diaphragm**							
Minute 1	1.50 cm/s	0.71 (0.54-0.88)	0.021	0.82 (0.60-0.95)	0.63 (0.38-0.84)	0.720	0.250
Minute 2	1.45 cm/s	0.72 (0.56-0.88)	0.015	0.70 (0.47-0.87)	0.63 (0.38-0.85)	0.696	0.368
Minute 3	1.55 cm/s	0.69 (0.52-0.87)	0.036	0.78 (0.56-0.93)	0.61 (0.36-0.83)	0.720	0.313

AUC, area under the curve; CI, confidence interval; PPV, positive predictive value; NPV, negative predictive value.

Figure E2. Repeated-measures correlations between peak transdiaphragmatic pressure (Pdi,peak), and diaphragm excursion (EXdi, upper panels), and peak contraction velocity (PCVdi, lower panels), in all patients (lefts panels), in weaning success patients (middle panels) and in weaning failure patients (right panels).

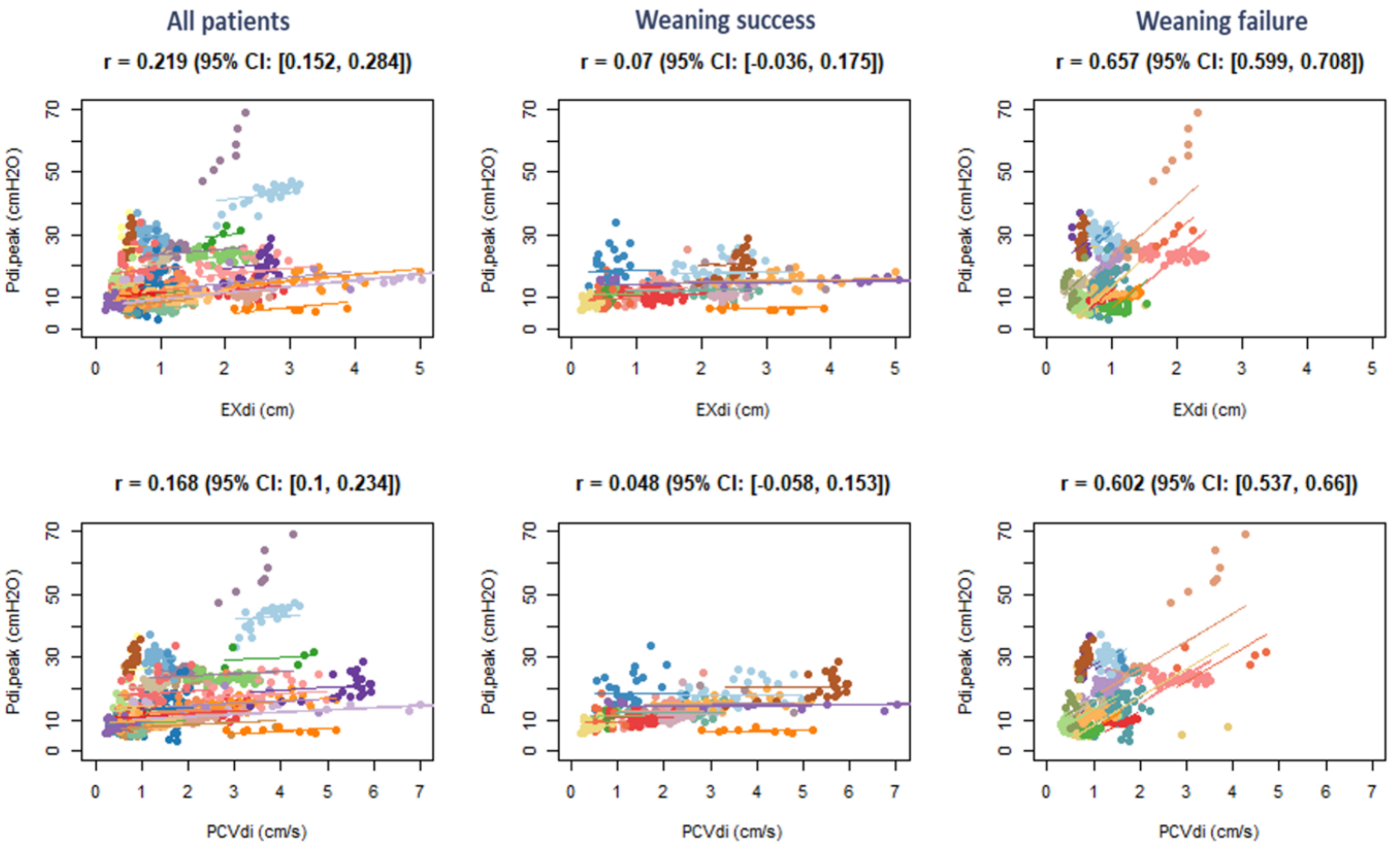

Figure E3. Intra-individual Spearman's correlations between transdiaphragmatic pressure-time product (PTPdi), and diaphragm excursion (EXdi, upper panels) and peak contraction velocity (PCVdi, lower panels), in all patients (left panels), in weaning success patients (middle panels) and in weaning failure patients (right panels). Solid line signifies the linear regression line.

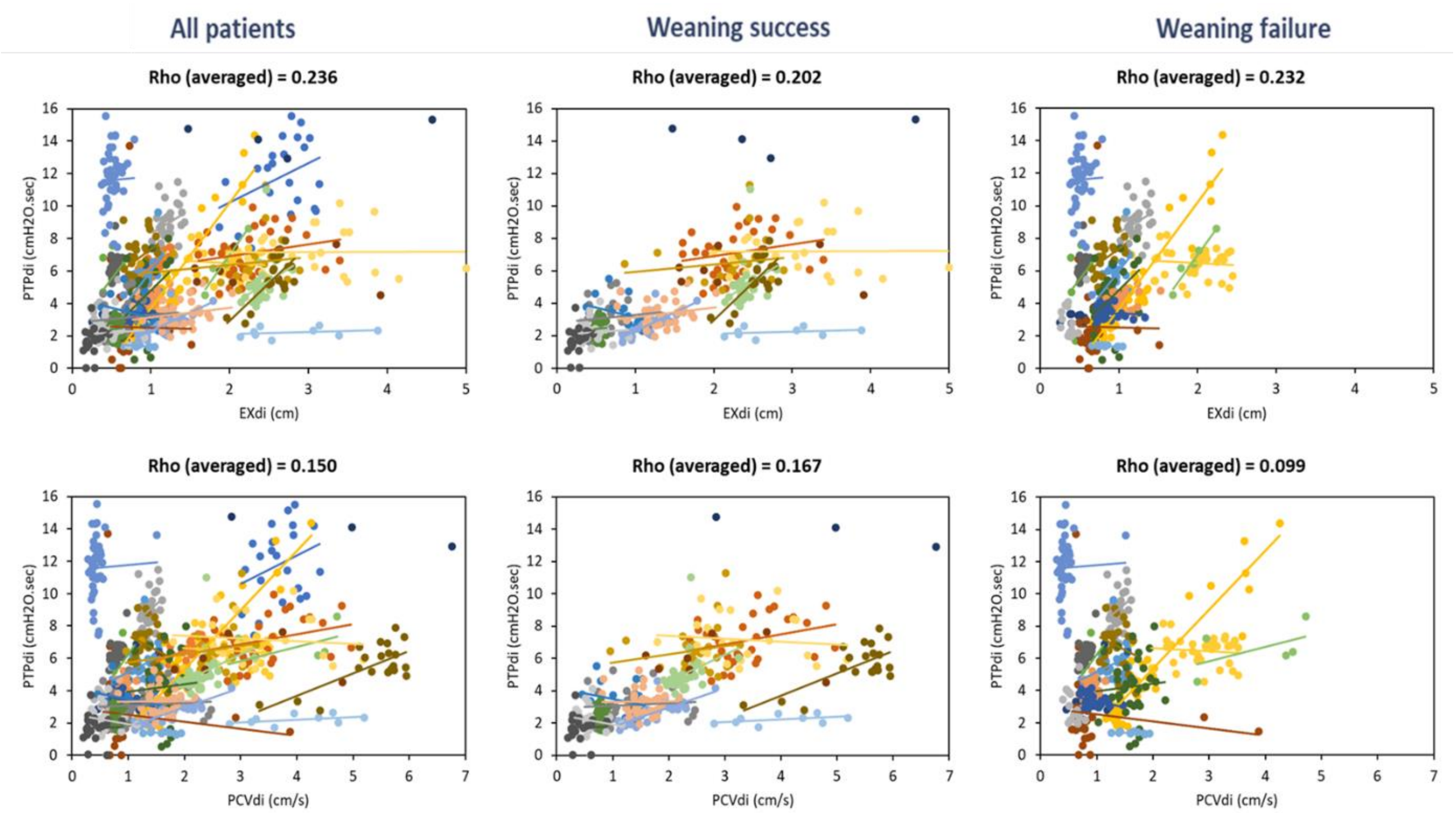

Figure E4. Intra-individual Spearman's correlations between peak transdiaphragmatic pressure (Pdi,peak), and diaphragm excursion (EXdi, upper panels) and peak contraction velocity (PCVdi, lower panels), in all patients (left panels), in weaning success patients (middle panels) and in weaning failure patients (right panels). Solid line signifies the linear regression line.

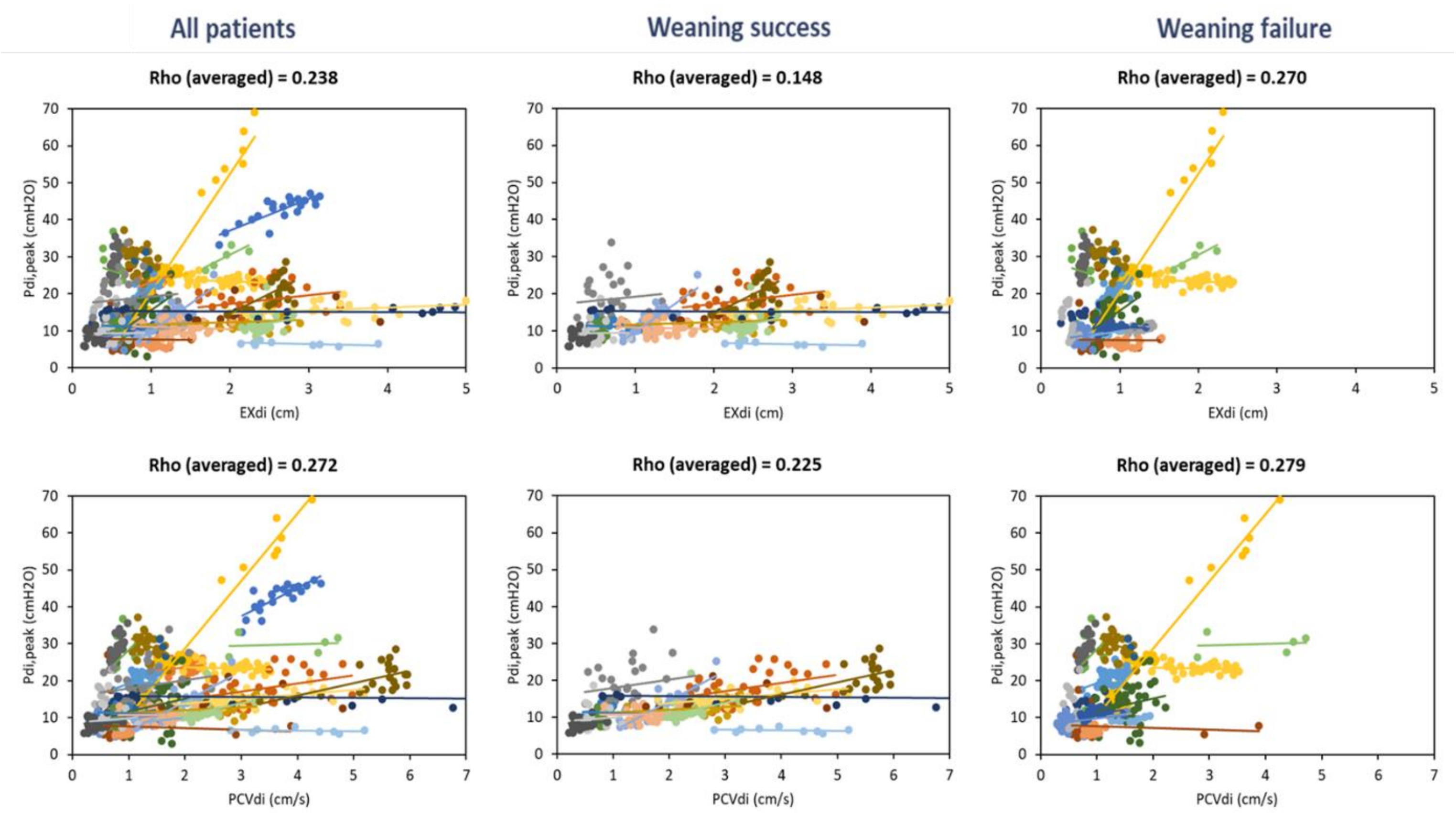

Table E3. Strength of intra-individual Spearman's correlation between transdiaphragmatic pressure-time product (PTPdi) or peak transdiaphragmatic pressure (Pdi,peak) and diaphragm excursion (EXdi) or peak contraction velocity (PCVdi).

0 – 0.19, "very weak" – dark red; 0.20 – 0.39 "weak" – light red; 0.40 – 0.59 "moderate" – yellow; 0.60 – 0.79 "strong" – light green; 0.80 – 1.0 "very strong" – dark green

Patient #	Weaning outcome	PTPdi vs. PCVdi	PTPdi vs. EXdi	Pdi,peak vs. PCVdi	Pdi,peak vs. EXdi
1	Failure	0.305	0.278	0.300	0.478
2	Failure	0.794	0.889	0.827	0.928
3	Failure	0.341	0.509	0.465	0.498
4	Failure	0.214	0.214	0.357	-0.179
5	Failure	0.100	0.117	0.733	0.733
6	Failure	-0.240	0.106	-0.081	0.133
7	Failure	0.468	0.426	0.763	0.586
8	Failure	-0.157	0.350	-0.415	-0.415
9	Failure	0.000	0.000	0.000	0.000
10	Failure	0.019	0.472	0.136	0.511
11	Failure	0.166	0.116	0.305	0.274
12	Failure	0.055	0.119	0.074	0.064
13	Failure	-0.288	-0.226	-0.147	-0.257
14	Failure	-0.065	-0.011	-0.121	-0.129
15	Failure	-0.479	-0.552	0.636	0.491
16	Failure	0.700	1.000	0.400	0.900
17	Failure	-0.258	0.141	0.510	-0.025
18	Success	0.322	0.317	0.332	0.300
19	Success	0.209	0.246	0.331	0.213
20	Success	0.160	0.250	0.196	0.297
21	Success	-0.272	-0.436	-0.025	-0.096
22	Success	0.125	0.089	0.188	0.156
23	Success	0.797	0.801	0.856	0.838
24	Success	0.079	0.179	-0.034	-0.090
25	Success	-0.093	0.124	-0.066	-0.242
26	Success	-0.178	-0.028	0.542	0.271
27	Success	0.450	0.317	-0.300	-0.400
28	Success	0.469	0.413	0.497	0.137
29	Success	0.038	0.084	0.221	0.269
30	Success	-0.071	-0.071	0.179	0.179
31	Success	0.132	0.325	0.002	-0.081
32	Success	0.342	0.417	0.458	0.464
33	N/A	0.239	0.280	0.819	0.773
34	N/A	0.662	0.785	0.312	0.502

Table E4. Direction of intra-individual Spearman's correlation between transdiaphragmatic pressure-time product (PTPdi) or peak transdiaphragmatic pressure (Pdi,peak) and diaphragm excursion (EXdi) or peak contraction velocity (PCVdi).

0.5 0– 1.0 – Dark green; 0.0 – 0.49 – Light green; -0.01 – -0.49 – Light red; -0.50 – -1.0 – Dark red

Patient #	Weaning outcome	PTPdi vs. PCVdi	PTPdi vs. EXdi	Pdi,peak vs. PCVdi	Pdi,peak vs. EXdi
1	Failure	0.305	0.278	0.300	0.478
2	Failure	0.794	0.889	0.827	0.928
3	Failure	0.341	0.509	0.465	0.498
4	Failure	0.214	0.214	0.357	-0.179
5	Failure	0.100	0.117	0.733	0.733
6	Failure	-0.240	0.106	-0.081	0.133
7	Failure	0.468	0.426	0.763	0.586
8	Failure	-0.157	0.350	-0.415	-0.415
9	Failure	0.000	0.000	0.000	0.000
10	Failure	0.019	0.472	0.136	0.511
11	Failure	0.166	0.116	0.305	0.274
12	Failure	0.055	0.119	0.074	0.064
13	Failure	-0.288	-0.226	-0.147	-0.257
14	Failure	-0.065	-0.011	-0.121	-0.129
15	Failure	-0.479	-0.552	0.636	0.491
16	Failure	0.700	1.000	0.400	0.900
17	Failure	-0.258	0.141	0.510	-0.025
18	Success	0.322	0.317	0.332	0.300
19	Success	0.209	0.246	0.331	0.213
20	Success	0.160	0.250	0.196	0.297
21	Success	-0.272	-0.436	-0.025	-0.096
22	Success	0.125	0.089	0.188	0.156
23	Success	0.797	0.801	0.856	0.838
24	Success	0.079	0.179	-0.034	-0.090
25	Success	-0.093	0.124	-0.066	-0.242
26	Success	-0.178	-0.028	0.542	0.271
27	Success	0.450	0.317	-0.300	-0.400
28	Success	0.469	0.413	0.497	0.137
29	Success	0.038	0.084	0.221	0.269
30	Success	-0.071	-0.071	0.179	0.179
31	Success	0.132	0.325	0.002	-0.081
32	Success	0.342	0.417	0.458	0.464
33	N/A	0.239	0.280	0.819	0.773
34	N/A	0.662	0.785	0.312	0.502
